# Outcomes in patients with severe sepsis or septic shock with a urinary source of infection in the ICU

**DOI:** 10.1186/2197-425X-3-S1-A84

**Published:** 2015-10-01

**Authors:** ML Pérez Pérez, A Ortega López, A Pérez Lucendo, B Lobo Valbuena, N Martínez Sanz, J Palamidessi Domínguez, R Fernandez Rivas, P Matía Almudevar, A Naharro Abellán, P Galdos Anuncibay

**Affiliations:** Hospital Universitario Puerta de Hierro Majadahonda, Madrid, Spain

## Introduction

Urinary tract infection (UTI) is a heterogeneous syndrome ranging from cystitis to bacteremia with shock and multiple organ failure.

## Objectives

To assess the characteristics of patients admitted to the ICU with a main diagnosis of UTI complicated with severe sepsis or septic shock. We focused on relevant urologic history (UH), radiological findings (RF), need of urologic procedures, causative organisms, antimicrobial therapy (AT) and patients' outcomes in the ICU.

## Methods

Retrospective observational study conducted in a medical ICU of a tertiary university hospital. Demographic data, model scores (APACHE, SOFA), UH, immunosuppression, RF, microorganisms, AT, number of organ failures (NOF), supportive care and overall mortality were recorded.

## Results

Fifty four patients with a urinary source of infection were admitted to the ICU between May 2012 and March 2015. Mean age 61.1 ± 13.6. Men 55.6%. 11 immunosuppressed patients. Mean APACHE II 17.2 ± 9.6. Mean SOFA 6.6 ± 3.1. First lactic acid 3.1 ± 2.1. Average length of stay 4.4 ± 2.1 days.

Fifty five point six of these patients had a previous UH: 22.2% needed Double J stents, 14.8% transrectal prostatic biopsy and 24.1% needed nephrostomy tubes or other procedures.

The main diagnosis on admission was acute pielonefritis (57.4%); followed by sepsis related to transrectal prostatic biopsy (14.8%), complicated urinary tract infection (11.1%) and prostatitis (7.4%).

We found RF in 43.7%. 27.4% needed urologic procedures during the ICU stay.

Antimicrobial resistance was detected among twenty two (40.7%) of the isolated microorganisms. *Escherichia coli* was the predominant microorganism: 14 were resistant (R) to ciprofloxacin, 2 R to cotrimoxazole and 4 presented extended-spectrum beta-lactamases. Carbapenems were used in 64.8% patients. After empiric antimicrobial therapy, we de-escalated in 66.7% of these patients, with no associated complications.

NOF were 1 or 2 in 74.1%: 70.4% needed vasoactive support, 4 patients required mechanical ventilation and 4 received continuous renal replacement therapy. 3 patients died (5.6%).

Patients with UH needed more urologic procedures and had more NOF, but no statistically significant differences were found {RR 1.9, IC 95% (0.5-6.5)}, {RR 2.5, IC 95% (0.69-9)}.

A statistical trend showed that patients with a higher number of RF required more urologic procedures, although with no statistical significance {RR 2.1, IC 95% (0.6 - 7.3)}. Furthermore, no statistical significance association was found between UH and bacterial resistance or immunosuppression.

## Conclusions

Patients with severe sepsis or septic shock due to UTI in the ICU had a satisfactory outcome.

De-escalation was done in two thirds of the patients with no complications.

We found a high percent of patients with a previous urologic history and radiological findings who needed more urologic procedures but no statistically significant associations were found.

